# Range of motion after total knee arthroplasty in hemophilic arthropathy

**DOI:** 10.1186/s12891-018-2080-0

**Published:** 2018-05-22

**Authors:** Radovan Kubeš, Peter Salaj, Rastislav Hromádka, Josef Včelák, Aleš Antonín Kuběna, Monika Frydrychová, Štěpán Magerský, Michal Burian, Martin Ošťádal, Jan Vaculik

**Affiliations:** 1 Department of Orthopaedics, 1st Faculty of Medicine, Charles University and Na Bulovce Hospital, Budínova 2, 180 81 Prague 8, Czech Republic; 2 Institute of Clinical and Experimental Hematology, First Faculty of Medicine, Charles University and Institute of Hematology and Blood Transfusion, U Nemocnice 1, 12802 Prague 2, Czech Republic; 3 Department of Orthopaedics, 1st Faculty of Medicine, Charles University and Motol University Hospital, Úvalu 84, 15006 Prague 5, Czech Republic; 4 Department of Social and Clinical Pharmacy, Faculty of Pharmacy, Charles University, Akademika Heyrovského 1203, 500 05 Hradec Králové, Czech Republic

**Keywords:** Hemophilic arthropathy, Total knee replacement, Range of motion, Flexion contracture, Hemophilia, Orthopaedics

## Abstract

**Background:**

Outcomes of total knee replacement in cases of hemophilic patients are worse than in patients who undergo operations due to osteoarthritis. Previous publications have reported varying rates of complications in hemophilic patients, such as infection and an unsatisfactory range of motion, which have influenced the survival of prostheses. Our retrospective study evaluated the data of hemophilic patients regarding changes in the development of the range of motion.

**Methods:**

The data and clinical outcomes of 72 total knee replacements in 45 patients with hemophilia types A and B were reviewed retrospectively. Patients were operated between 1998 and 2013. All of the patients were systematically followed up to record the range of motion and other parameters before and after surgery.

**Results:**

The mean preoperative flexion contracture was 17° ± 11° (range, 0°-40°), and it was 7° ± 12° (range, 0°-60°) postoperatively. The mean flexion of the knee was 73° ± 30° (range, 5°-135°) before the operation and 80° ± 19° (range, 30°-110°) at the last follow-up. The mean range of motion was 56° ± 34° (range, 0°-130°) before the operation and 73° ± 24° (range, 10°-110°) at the last follow-up.

**Conclusions:**

Statistical analysis suggested that the range of motion could be improved until the 9th postoperative week. The patient should be operated on until the flexion contracture reaches 22° to obtain a contracture < 15° postoperatively or until the contracture reaches 12° to obtain less than 5°. The operation generally does not change the flexion of the knee in cases of hemophilic patients, but it reduces the flexion contracture and therefore improves the range.

## Background

The knee is the most commonly impaired joint in cases of patients with hereditary bleeding disorders [1]. Hemophilic patients have a reduced quality of life due to cartilage and bone damage, which causes loss of mobility. The pathological processes inside the joints remain subjects of different theories and remain unclear. Several papers have described good functional results and reduction of these problems after total knee arthroplasty (TKA). Previous reports have reported varying rate of complications, such as infection and flexion contracture, and survival [2–12] of TKA, which are much more frequent than in patient with osteoarthritis [1, 13, 14].

**Fig. 1:**
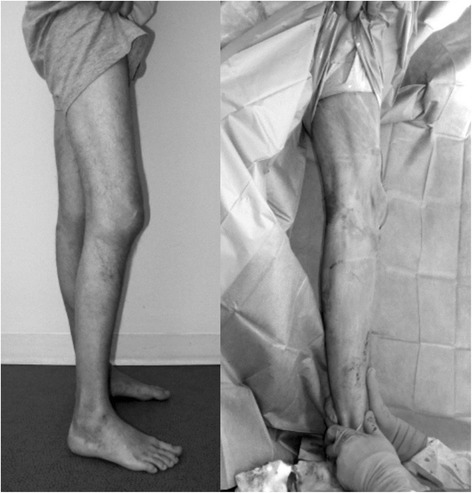
Clinical view of the same knee - preoperative extension deficiency and its correction after implantation of the TKR

One of the most important parameters of the outcomes of surgery with a close correlation with quality of life (QOL) is the range of motion (ROM). In particular, extension of the knee, i.e., the level of postoperative flexion contracture, is important for the mobility of the patient [13]. Flexion contracture affects the function of the operated joint, but it also impairs the gait and the day living activities (Fig. 1).

This retrospective study evaluated the data from hemophilic patients regarding the range of motion. The aim of this study was to provide information about preoperative and postoperative parameters that can delineate the functional outcomes of TKA. Parameters, such as flexion contracture, flexion and range of motion, were followed before and after implantation to provide detailed information about their development.

## Methods

The data and clinical outcomes of 72 TKAs in 45 patients with hemophilia A and B were reviewed retrospectively. The patients were operated on over a period of 15 years between 1998 and 2013 by one surgeon (RK). The main objective criteria for surgery were recurrent bleeding conditions, pain and difficult mobility in normal daily activities or a rapid progression of flexion contracture and the subjective will of the patient to improve the QOL. However, all of the patients were strongly warned that TKR has a limited survival rate, and all of the patients had to sign an informed consent form.

Preoperatively, hematological and biochemical analyses were performed. The hematological status, grade of failure, and type of coagulation disorder were known from the hematologic history. Everything was checked prior to the operation to find any changes, especially regarding the level of coagulation activity and the detection and level of inhibitors. The traditional classification of hemophilia severity was used to distribute the patients into three groups: severe (less than 1% activity of the factor), moderate (1 to 5%) and mild (5 to 40%) [1, 15]. Distribution of our patients is shown in Table 1. The status of HIV and hepatitis activity was determined in every case.

**Table 1:** Number of patients according severity of haemophilia

		Hemophilia A	Hemophilia B
No. of Patients		42	3
Severity	Severe	24	1
	Moderate	16	2
	Mild	2	–
No. of TKA		67	5

**Fig. 2:**
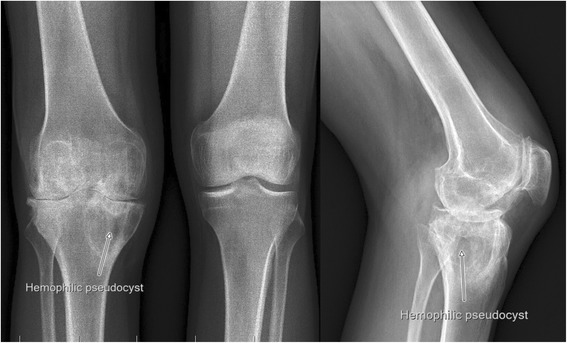
Preoperative X-ray of the typical hemophiliac arthropathy of the right knee with hemophiliac pseudocyst of the proximal tibia

Management before the surgery involved assessing the nature and frequency of bleeding and the patient history taken by the surgeon. The following parameters were evaluated on follow-ups: recurrence of bleeding episodes into the joint and physical examination of range of motion (ROM), i.e. extension (flexion contracture) and flexion. X-rays of the joint were routinely obtained (Fig. 2). All of the patients included in the study were severely affected with recurrent hemarthroses of the knee joint. The Knee Society score [16] was used for evaluation of patients before and after the operations at every follow up.

The hematologic part of the operation was fully under the control of our cooperating hematologic department (Institute of Clinical and Experimental Hematology, UHKT); in the most difficult cases, a hematologist was also a member of our operation group and cooperated with the anesthesiologist.

Currently, we prefer the model of a short preoperative rehabilitation program under full coverage of prophylactic therapy to prevent any bleeding close to surgery and improve muscle function and prepare patients for postoperative activity. We know that the first postoperative days are usually very painful, and we cannot expect much muscle activity. This physical therapy (PT) is done in our cooperating hematologic department (UHKT) and helps to predict the postoperative behavior of our patients.

The preoperative examination standardly involved physical and X-ray evaluation of all of the large joints, i.e. the shoulder, elbow, hip, knee and ankle joints which facilitated the planning of multiple operations. In these cases, we always considered that postoperative PT programs and, especially, walking activity were very important to the status of the upper extremities for the possibility of walking with the help of crutches, especially in cases of bilateral procedures.

The standard implantation of TKA involved mid-line incision and a medial parapatellar approach. A tourniquet is usually used only in the phase of the procedure when all resections are done and gaps are balanced - i.e. just before implantation (cementing) when we clean and “dry” (ideally with the help of “pulsation” jet lavage) resected surfaces.

The limited time of tourniquet use strategy, according to our recommendation, has these reasons:

- Usually very complicated bone and soft tissue status prevents to finish operation during 60 min.
- Meticulous hemostasis is possible and fully advisable - esp. during release of soft tissue adhesions.
- In cases of release of soft tissue flexion contracture, when we manipulate full extension, we could cause traction tears of the popliteal vessels, which could be immediately diagnosed and solved.

This strategy also has the disadvantage of increased blood loss, both initially and overall, and worse initial visibility of the operation field, but gives less risk of immediate postoperative bleeding complications for your patients.

**Fig. 3:**
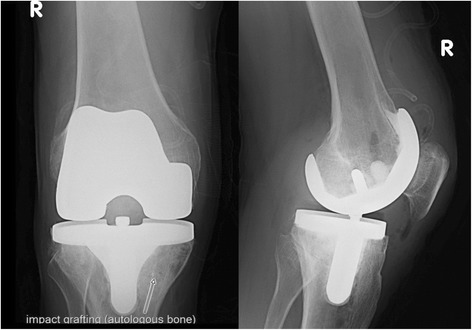
Postoperative X-ray of the same knee with impact grafting of the haemophiliac pseudocyst (cemented standard CR implant)

All types of cemented TKA implants were used, according to the type of impairment of the knee. Standard cruciate retaining or posterior stabilized implants were implanted, and in 7 cases, revision TKA or hinged knees implants for primary implantation were used (Fig. 3). The patients with all types of implants were used as a single group for the statistics. Separated groups of patients were statistically not significant when compare to each other. Second-generation cephalosporin was used as antibiotic prophylaxis, with one dose administered before and 2 doses after the operation.

Unilateral TKA or bilateral TKA was performed with a single admission in one or two procedures. Bilateral operations were performed in 18 patients (36 TKAs). The procedures were performed in parallel or sequentially on both knees at a single procedure in these cases. In 8 cases, hip replacement was performed sequential to knee replacement. In 9 patients, both knees were operated on (18 TKAs), but the TKAs were implanted in two admissions. The patients were discharged from hospital when finish postoperative protocol of the physiotherapy and their haemophilia was stabilized.

Patients were assessed at follow-ups at 6 weeks, 3 months, 6 months, and 12 months and then every 12 months after discharge from the hospital. X-rays were obtained routinely at each follow-up, and the ROM of the operated knee joint (joints) was measured. The Knee Society score was evaluated at every follow-up.

## Results

The median age of the patients at the time of the operation was 47.4 ± 13.3 years (range, 35–55). The patients were followed up for a median of 8.9 ± 4.3 years (range 6.3–13.1). The median time of hospitalization of a patient after the TKA surgery was 30 ± 12.5 days (range, 24–36 days).

**Fig. 4:**
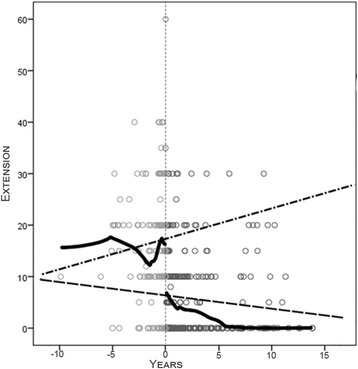
The extension of the knee before and after the implantation of TKA. Lines represent increasing tendency (dash-dotted line) of the average value of preoperative flexion contracture and postoperative decrease in the parameter (dash line). Irregular solid curves show the medians of the parameters pre- and postoperatively. Small circles represent measured values

**Fig. 5:**
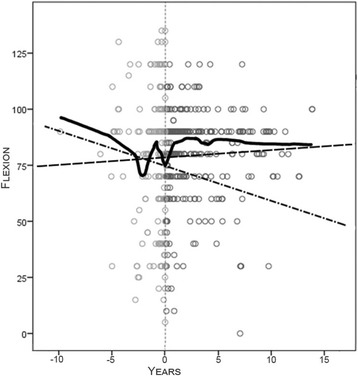
The flexion of the knee before and after the implantation of TKA. Lines represent the decreasing tendency of the average value of preoperative flexion (dash-dotted line) contracture and the postoperative increase in the parameter (dash line). The irregular solid curve shows average values of the parameter pre- and postoperatively. Small circles represent measured values

The mean preoperative flexion contracture was 17° ± 11° (median 15°, range 0°- 40°), and it was 7° ± 12° (median 0°, range 0° to 60°) postoperatively (Fig. 4). The mean flexion of the knee was 73° ± 30° (range 5°- 135°) before the operation and 80° ± 19° (range 30°- 110°) at the last follow up - Fig. 5. The mean ROM was 56° ± 34° (range 0° - 130°) before the operation and 73° ± 24° (range 10°- 110°) at the last follow up.

The Knee Society Score [16] was used in all 72 cases to evaluate the outcomes of TKA before and after the operation. The mean clinical score before surgery was 28 (range, 13–36), and after TKA at the last follow up, it was 72 (range 62 to 80). The average functional score before the operation was 37 (range 15 to 50), and after the operation, it was 70 (range 50 to 80) in Table 2.

**Table 2:** The Knee Society Score; outcomes of 72 TKA before and after the operation

	Age	Clinic Score		Functional Score	
		Before	After	Before	After
Score	41	28	72	37	70
(Range of Score)	30–62	13–36	68–80	15–50	50–80

Complications were evaluated retrospectively according to the standardized list of the Knee Society [17]. Deep infection, superficial infection and loosening of the implants were recorded in 9 cases (12.5%). We did not register any case of nerve palsy, bleeding or vascular injury or other complications associated with TKA in this study. There was no occurrence of the development of clotting factor inhibitors in reviewing this group of patients.

In the study, 2 patients had proven HIV. The patients acquired the virus from infected clotting factors before the beginning of the study.

## Discussion

The surgery for TKA in a patient with hemophilia is a complex procedure. The outcomes of the surgery depend of many factors. Typical for a patient with hemophilia is the progressive deterioration of the knee joint after bleeding episodes, which leads to a restriction of the joint’s range of motion. Although the main goal of the TKA is to reduce pain, the range of motion is important for satisfactory outcomes.

Patients with hemophilia and severe knee arthritis frequently present with tree plane knee deformities [13]. The typical deformity is flexion on the sagittal plane, with contracture and external rotation. Destruction of joint surfaces on the lateral side leads to valgus deformity and dorsal subluxation of the tibia, which limit the of range of motion and cause fibrosis of the surrounding soft tissue. These deformities, together with the presence of periarticular osteopenia and cysts, cause postoperative flexion contractures [13, 18] (Figs. 1, 2). The average range of motion of hemophilic patients after total knee replacement is less than that in cases of patients with osteoarthritis. Intra-capsular fibrotic changes and extra-capsular muscle contractures are typical problems that affect the operative outcomes after TKA [19]. The attention in preoperative planning should be focused on flexion contracture [13].

The flexion contracture of the knee joint, especially its rapid progression, is one of the important indication criteria for TKA surgery. The level of postoperative contracture is important for successful outcomes of surgery as well. Goddard [1] reported that the contracture improved from 18° (range, 0° to 50°) to 8° (range, 0° to 30°) at the time of the latest follow-up. Bae [18] published an average flexion contracture of 22.7° before implantation and 5.7° after surgery. Ernstbrunner et al. [11] published an average flexion contracture of 18° before implantation and 6° after operation. Atilla [13] argued that 27.5° preoperative flexion contracture led to extensive flexion contracture of 15° postoperatively, which significantly affected the gait. The operation in hemophiliacs should be not postponed when the contracture level reaches 15° [13].

In our study, the operation was usually performed due to the rapid progression of flexion contracture within the last year before implantation. We retrospectively and systematically evaluated the residual lack of extension. The flexion contracture develops in association with bleeding episodes. The graph (Fig. 4) shows the values of extension limitations. The average value of contracture generally tends to increase by 0.95° per year until surgery. The figure shows that the median contracture in cases of hemophilic patients slowly decreases over the years, but then it suddenly changes its course and increases. The operation should be planned with regard to changing the course of development of the contracture.

The contracture decreases rapidly in the first two months after TKA surgery and then significantly slows. Although the average lack of extension in our study was 7° at the last follow up, the median was 0° overall. The tendency of this parameter is to decrease after surgery, as shown in Fig. 1.

The flexion tended to decrease over time preoperatively in all cases over years (Fig. 5). Evaluation of the data showed a paradoxical increase in flexion, while the lack of the extension increased several months before surgery. After surgery, the flexion did not change significantly (*p* = 0.435). The profit was only 7° at the last follow up in the group of patients. A patient could not expect better flexion of the joint, but the pain was decreased [20]. The patient could expect an improvement of ambulation because the surgery improved the ability to extend the joint [13]. Song [10] et al. reported average improvement of the flexion 4.9° after the operation.

The ROM of the knee was calculated as the subtraction of the extension from the flexion in our study. Goddard [1] published that the ROM was 68° (20° to 130°) preoperatively and 79° (20° to 120°) at the final follow-up. Bae [18] reported that the average preoperative range of motion was 73.4°, whereas the average postoperative range of motion increased to 92.3°. Atilla [13] reported an average range of motion of 37.6°, which improved to 57.1° postoperatively. All of these studies reported only a slight improvement in ROM. In our study, the pre- and postoperative ROMs were comparable to these results, and the average total flexion arc improved from 56° to 73°.

The postoperative range of motion compared to TKR in non-hemophilic patients is more limited. The implantation of TKA mostly affects and reduces the flexion contracture. The ROM of the knee improves, mostly due to the decrease in the flexion contracture. The operation significantly (*p* < 0.001) increased of the ROM, and the increase in this parameter was 17° in this group of patients.

The implantation affects the ROM, but often, the flexion contracture persists at some level. A contracture of the lower extremity joint affects the gait pattern [21]. Atilla et al. [13] reported that a flexion contracture of < 15° of the knee is satisfactory in cases of hemophilic patients. In this group of patients, we attempted to propose a threshold for the operation on the affected knee joint to reach a satisfactory level of contracture. The evaluation of the data showed that preoperative flexion contracture < 22° led to postoperative contracture < 15°, and preoperative contracture < 12° resulted in postoperative contracture < 5°. In our group, the rates were 72 and 50% of patients, respectively. We believe that TKA should be implanted until the flexion contracture is 22°.

The outcomes of the operations were evaluated by the Knee Society functional score (KSS) in our study Tab. 2. The clinical part of the KSS showed an average improvement of 44 points. The patients demonstrated satisfactory pain control because all of the patients rated the pain as better than moderate and improved. The suboptimal improvement in motion (see above) associated with satisfactory reduction of the knee pain was acceptable for most of the patients with hemophilia. The limited flexion was balanced by improvement of the flexion contracture. The average improvement in the functional score was 33 points. None of the patients referred to being housebound.

The outcomes were comparable with other studies. Bae [18] reported that the average preoperative knee score increased from 18.6 points (range, 3–29) to 82.8 points (range, 44–99). The average preoperative knee function score increased from 41.4 points (range, 20–60 points) to 75.8 points (range, 45–95 points). Goddard [1] used the HSS score in 49 patients (60 TKRs). The mean clinical score after surgery was 82 (70 to 95), and 95% of the TKAs had good or excellent results. Ernstbrunner [11] reported that the preoperative average clinical and the functional knee score increased from 36 and 62 points to 73 and 78 points after the operation, respectively.

## Conclusion

Total knee replacement improves the mobility of hemophilic patients, especially the functional range of motion, and it provides satisfactory pain relief. The operation generally does not change the flexion of the knee, but it reduces the flexion contracture and therefore improves the range of motion. Statistical analysis suggested that the range of motion could be improved until the ninth postoperative week.

## Abbreviations

PT: physical therapy
QOL: Quality of life
ROM: Range of motion
TKA: Total knee arthroplasty
